# Maize ZmFNSI Homologs Interact with an NLR Protein to Modulate Hypersensitive Response

**DOI:** 10.3390/ijms21072529

**Published:** 2020-04-05

**Authors:** Yu-Xiu Zhu, Chunxia Ge, Shijun Ma, Xiao-Ying Liu, Mengjie Liu, Yang Sun, Guan-Feng Wang

**Affiliations:** The Key Laboratory of Plant Development and Environmental Adaptation Biology, Ministry of Education, School of Life Sciences, Shandong University, Qingdao 266237, China; 18754805371@163.com (Y.-X.Z.); chunxia_ge@126.com (C.G.); mazidong123go@sina.com (S.M.); 17862950676@163.com (X.-Y.L.); mjliu1988@163.com (M.L.); yangsun1985@126.com (Y.S.)

**Keywords:** plant innate immunity, NLR, disease resistance, FNSI, hypersensitive response, maize

## Abstract

Nucleotide binding, leucine-rich-repeat (NLR) proteins are the major class of resistance (R) proteins used by plants to defend against pathogen infection. The recognition between NLRs and their cognate pathogen effectors usually triggers a rapid localized cell death, termed the hypersensitive response (HR). Flavone synthase I (FNSI) is one of the key enzymes in the flavone biosynthesis pathway. It also displays salicylic acid (SA) 5-hydroxylase (S5H) activity. A close homolog of FNSI/S5H displays SA 3-hydroxylase (S3H) activity. Both FNSI/S5H and S3H play important roles in plant innate immunity. However, the underlying molecular mechanisms and the relationship between S5H and S3H with the NLR-mediated HR are not known in any plant species. In this study, we identified three genes encoding ZmFNSI-1, ZmFNSI-2 and ZmS3H that are significantly upregulated in a maize line carrying an autoactive NLR *Rp1-D21* mutant. Functional analysis showed that ZmFNSI-1 and ZmFNSI-2, but not ZmS3H, suppressed HR conferred by Rp1-D21 and its signaling domain CC_D21_ when transiently expressed in *N. benthamiana*. ZmFNSI-1 and ZmFNSI-2 physically interacted with CC_D21_. Furthermore, ZmFNSI-1 and ZmFNSI-2 interacted with HCT, a key enzyme in lignin biosynthesis pathway, which can also suppress Rp1-D21-mediated HR. These results lay the foundation for the further functional analysis of the roles of FNSI in plant innate immunity.

## 1. Introduction

To protect against the invasion of different pathogens, plants have evolved multiple sophisticated strategies including pathogen-associated molecular pattern (PAMP)-triggered immunity (PTI) and effector-triggered immunity (ETI) [1,2]. ETI is activated when plant disease resistance (*R*) genes recognize the presence of specific pathogen-secreted molecules (effectors), triggering a series of defense responses which often include a localized programmed cell death at the point of pathogen challenge known as the hypersensitive response (HR) [3,4,5]. The HR can restrict the further expansion of pathogenic microorganisms and therefore is usually regarded as a hallmark for plant disease resistance [4,6,7].

Most *R*-genes encode so-called NLR proteins, which contain a central nucleotide-binding (NB) domain and a C-terminal leucine-rich repeat (LRR) domain [8,9]. The NLR proteins can be further divided into two major classes according to their N-terminal domains; CC-NLRs (CNLs) contain a predicted coiled-coil (CC) domain and TIR-NLRs (TNLs) carry an N-terminal homology to the intracellular Toll-IL1 receptors (TIRs) domain [9,10]. TNLs are restricted to dicots while CNLs have been found in both monocots and dicots.

The *Rp1* locus on maize chromosome 10 carries multiple tandemly repeated CNL paralogs, one of which, *Rp1-D*, confers resistance to maize common rust caused by specific races of the fungus *Puccini sorghi* [11]. A high frequency of unequal crossovers has been observed between different paralogs at this locus [12]. The chimeric gene *Rp1-D21* mutant was formed by intragenic recombination between two paralogs, *Rp1-D* and *Rp1-dp2* [13,14]. *Rp1-D21* confers a dominant spontaneous HR lesion phenotype on leaves and stalks in the absence of pathogen infection [13,14]. The severity of this HR phenotype is affected by light, temperature, developmental stage and genetic background [15,16].

*Rp1-D21* has been used as a tool to identify loci, genes and pathways associated with modulation of the severity of HR [16,17,18,19]. Two genes encoding homologs of enzymes in lignin biosynthesis pathway, hydroxycinnamoyltransferase (HCT1806) and caffeoyl-CoA O-methyltransferase (CCoAOMT2), were shown to physically interact with and suppress the HR caused by Rp1-D21 [20,21]. Both lignin content and transcript abundance of the genes in the lignin biosynthesis pathway were increased in *Rp1-D21* compared to a near-isogenic wild type sibling [21], suggesting that the lignin biosynthesis pathway plays important roles in the Rp1-D21-mediated defense response.

The flavonoid biosynthesis pathway also plays important roles in plant disease resistance [22,23,24]. Flavonoids can be divided into several classes: flavanones, flavanols, flavonols, anthocyanins, isoflavones and flavones [25]. Flavones act as signal molecules in the establishment of symbiotic relationships between root nodulation bacteria and their leguminous plant hosts [26]. It showed that many genes in lignin, flavonoid and terpenoid biosynthesis pathways are upregulated in maize lines inoculated by *Fusarium verticillioides*, a fungus causing ear rot and stalk rot in maize [27]. Flavone synthase I (FNSI) catalyzes the conversion of the flavanones into the corresponding flavones [28]. It belongs to the 2-oxoglutarate Fe (II) dependent dioxygenase (2OGD) superfamily [29,30], which in turn can be divided into three subfamilies according to phylogenetic analysis: DOXA, DOXB and DOXC [30,31,32]. Genes in the DOXC subfamily, the largest subfamily, are involved in the biosynthesis of secondary metabolites, and include FNSI, flavanone 3-hydroxylase (F3H), anthocyanidin synthase (ANS), and 1-aminocyclopropane carboxylic acid oxidase (ACO) [31].

Maize ZmFNSI-1 was demonstrated to be capable of converting the flavanones naringenin and eriodictyol into their corresponding flavones, apigenin and luteolin, respectively in plants [28]. Two rice OsFNSI homologs were also reported to have FNSI activity by converting naringenin into apigenin [33,34]. Transgenic over-expression of ZmFNSI-1 in *Arabidopsis* causes increased levels of apigenin [28]. The *Arabidopsis* ZmFNSI homolog, DOWNY MILDEW RESISTANT 6 (AtDMR6, also named AtS5H) displays both FNSI activity and salicylic acid (SA) 5-hydroxylase (S5H) activity, catalyzing the formation of 2,5-DHBA by hydroxylating salicylic acid (SA) [28,29,35,36]. The *Atdmr6* mutant confers resistance to multiple pathogens, including *Hyaloperonospora parasitica*, *H. arabidopsidis*, *Phytophthora capsici* and *Pseudomonas syringae*. Expressing ZmFNSI in *Atdmr6* restores the susceptibility to these pathogens [28,29,36]. The *Atdmr6* mutant also accumulates higher levels of SA than wild type plants due to the S5H activity of AtDMR6 [35], which is important for mediating SA homeostasis during leaf senescence and pathogen responses. In *Arabidopsis*, DMR6-Like Oxygenase 1 (DLO1, also known as SA 3-hydroxylase, S3H) and DLO2, with high similarity with AtDMR6, negatively regulate the defense response to downy mildew and *P. syringae* pv. *tomato* (*Pst*) DC3000 [36,37]. Overexpression of DLO1/S3H and DLO2 in the *Atdmr6* mutant restores the susceptibility to downy mildew, indicating their partially redundant function with AtDMR6 [36]. The molecular mechanisms by which FNS1/S5H/DMR6 and S3H/DLO1 act in plant disease resistance are poorly understood and the relationship between FNSI and S3H with the NLR-mediated defense response is still unknown.

The aim of the study was to investigate the function of ZmFNSIs and ZmS3H in NLR protein Rp1-D21-mediated HR. We identified three genes encoding ZmFNSI-1, ZmFNSI-2 and ZmS3H that were significantly upregulated in *Rp1-D21* compared to the near-isogenic wild type line from RNA-seq analysis. We examined their roles in modulating Rp1-D21-mediated HR. ZmFNSI-1 and ZmFNSI-2, but not ZmS3H, suppressed Rp1-D21-mediated HR when transiently co-expressed with Rp1-D21 in *N. benthamiana*, however, they had no obvious effect on cell death caused by other elicitors. ZmFNSI-1 and ZmFNSI-2 physically interacted with the Rp1 protein as determined by coimmunoprecipitation (Co-IP) and yeast two hybrid (Y2H) assays. Furthermore, we showed that ZmFNSI-1 and ZmFNSI-2 interacted with HCT, which can also suppress Rp1-D21-mediated HR. We discuss the importance of FNSI in modulating plant defense response and lay the foundation for the further functional analysis of the roles in plant innate immunity.

## 2. Results

### 2.1. The Genes Encoding ZmFNSI and ZmS3H Are Induced in Rp1-D21 Compared to the Corresponding Wild Type

We have previously performed RNA-seq analysis to compare a near-isogenic pair of lines with and without *Rp1-D21* in two hybrid backgrounds, B73 × H95 and Mo17 × H95 [21]. We demonstrated that the genes encoding HCT1806, HCT4918 and CCoAOMT2 in the lignin biosynthesis pathway were highly induced in *Rp1-D21* compared to the corresponding wild type, and all of them suppressed Rp1-D21-mediated HR when transiently expressed in *N. benthamiana* [20,21].

**Figure 1:**
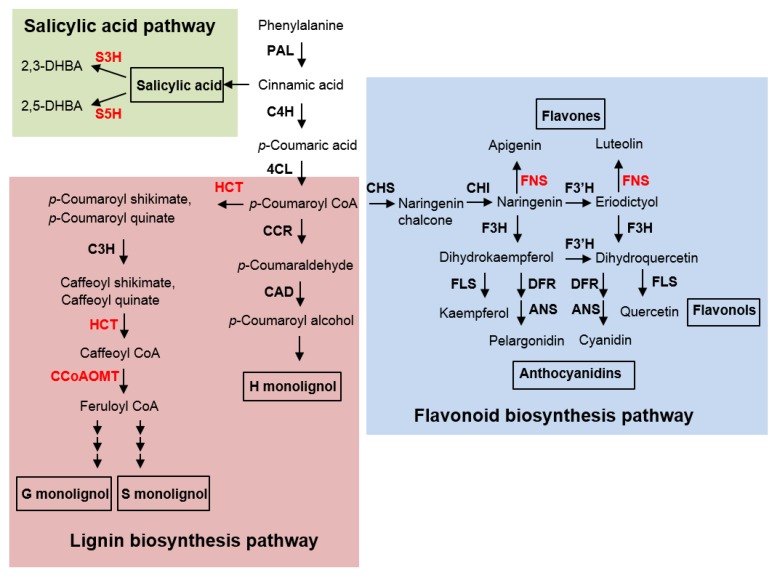
The phenylpropanoid pathway, modified according to previous studies [21,28]. 4CL, 4-hydroxycinnamoyl-CoA ligase; ANS, anthocyanidin synthase; C3H, *p*-coumarate 3-hydroxylase; C4H, cinnamate 4-hydroxylase; CAD, cinnamyl-alcohol dehydrogenase; CCoAOMT, caffeoyl-CoA *O*-methyltransferase; CCR, cinnamoyl-CoA reductase; CHI, halcone isomerase; CHS, chalcone synthase; COMT, caffeic/5-hydroxyferulic acid *O*-methyltransferase; DFR, dihydroflavonols reductase; F3′H, flavonol 3′-hydroxylase; F3H, flavanone 3-hydroxylase; F5H, ferulate 5-hydroxylase; FLS, flavonol synthase; FNSI, flavone synthase Ι; HCT, hydroxycinnamoyltransferase; PAL, phenylalanine ammonia lyase; S3H, SA 3-hydroxylase; S5H, SA 5-hydroxylase.

To further identify the genes modulating Rp1-D21-mediated HR or the plant defense response, we selected additional 19 genes that were upregulated in plants carrying *Rp1-D21* compared to their wild type counterparts and which had been otherwise implicated in plant disease resistance based on the literature for an initial screen. These genes included ZmFNSIs, ZmS3H, peptidoglycan-related genes, wall associated kinase, auxin-responsive genes, calmodulin binding proteins, etc. (Table 1). When transiently expressed in *N. benthamiana*, eleven of them had no obvious effect on Rp1-D21-mediated HR, two of them enhanced Rp1-D21-mediated HR and three of them showed autoactive HR, and we therefore concentrated on three genes related to ZmFNSI/ZmS3H that gave the most promising results. FNSI displays both FNSI activity by catalyzing the conversion of flavanones into flavones and S5H activity by hydroxylating SA into 2,5-DHBA, while S3H is able to hydroxylate SA into 2,3-DHBA (Figure 1).

**Figure 2:**
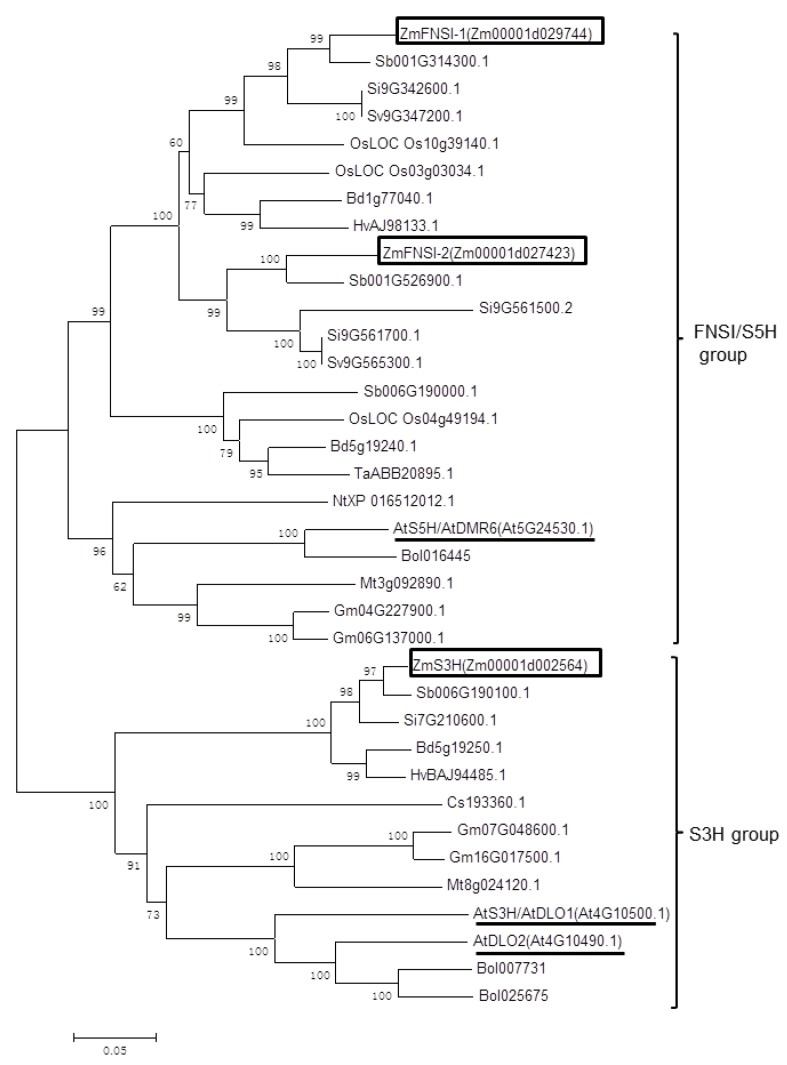
Phylogenetic analysis of FNSI/S5H and S3H proteins from different plant species. The protein sequences were aligned using Clustal X v2.1 and the phylogenetic tree was constructed using MEGA 6.0 software. At: *Arabidopsis thaliana*; Bd: *Brachypodium distachyon*; Bol: *Brassica oleracea capitata*; Cs: *Cucumis sativus*; Dc: *Daucus carota*; Gm: *Glycine max*; Hv: *Hordeum vulgare*; Mt: *Medicago truncatula*; Nt: *Nicotiana tabacum*; Os: *Oryza sativa*; Sb: *Sorghum bicolor*; Si: *Setaria italica*; Sv: *Setaria viridis*; Ta: *Triticum aestivum*; Tc: *Theobroma cacao*; Zm: *Zea mays.* The boxes and the underlines indicated the proteins from maize and *Arabidopsis*, respectively.

The *Arabidopsis* genome contains one AtDMR6/AtS5H/AtFNSI and two DMR6-like oxygenases, AtDLO1 (AtS3H) and AtDLO2. Using AtDMR6 and AtS3H as queries to blast the maize genome database (MaizeGDB), we identified two ZmFNSI homologs, ZmFNSI-1 (Zm00001d029744) and ZmFNSI-2 (Zm00001d027423), and one ZmS3H homolog (Zm00001d002564) in maize (Table 1). Phylogenetic analysis showed that FNSIs and S3Hs from different plant species are divided into two groups, and each group was further separated into two clades containing FNSIs or S3Hs from dicots and monocots (Figure 2).

We observed that the transcript levels of *ZmFNSI-1*, *ZmFNS1-2* and *ZmS3H* were highly induced in the *Rp1-D21* mutants compared to the corresponding wild type plants, with the transcript levels of *ZmFNSI-1* and *ZmFNSI-2* showing higher fold change (17.2 to 235 fold) than *ZmS3H* (4.2–8.6 fold) (Table 1).

### 2.2. ZmFNSI-1 and ZmFNSI-2 Suppress Rp1-D21-Mediated HR in N. benthamiana

**Figure 3:**
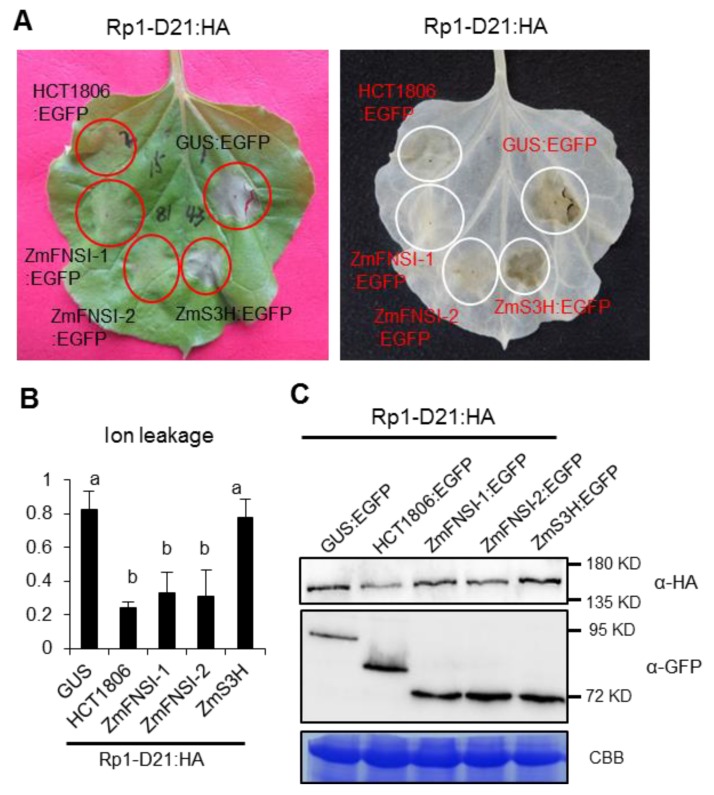
Investigating the function of ZmFNSIs and ZmS3H in Rp1-D21-induced HR. (**A**) ZmFNSIs and ZmS3H were transiently co-expressed with Rp1-D21 into *N. benthamiana*. The representative leaf was photographed at 3 days after inoculation (left), and the same leaf was cleared by ethanol (right). (**B**) Ion leakage conductivity (average ± standard error (SE), *n* > 5) was measured at 61 h after co-expression of GUS, HCT, ZmFNSIs or ZmS3H with Rp1-D21. Significant differences (*p* < 0.05) between samples are indicated by different letters (a–b). The protocol was measured according to our previous study [38]. (**C**) Total protein was extracted from agro-infiltrated leaves at 30 hpi. Anti-HA was used to detect the expression of Rp1-D21, and anti-GFP was used to detect the expression of GUS, HCT, ZmFNSIs and ZmS3H. Equal loading of protein samples was shown by Coomassie brilliant blue (CBB) staining of Rubisco.

To investigate the function of ZmFNSIs and ZmS3H in Rp1-D21-mediated HR, we employed the agrobacteria-mediated transient expression system in *N. benthamiana*. Rp1-D21 was fused with a 3× hemagglutinin tag at the C-terminus (Rp1-D21:HA), while ZmFNSI-1, ZmFNSI-2 and ZmS3H were fused with enhanced green fluorescent protein (EGFP) at the C-terminus (ZmS5H:EGFP and ZmS3H:EGFP). GUS:EGFP and HCT1806:EGFP were used as controls. Consistent with our previous studies [20,21,38], when GUS:EGFP was transiently co-expressed with Rp1-D21:HA in *N. benthamiana*, an obvious HR phenotype was observed at three days post-infiltration (dpi). No HR was observed in leaf sectors in which HCT1806:EGFP and Rp1-D21:HA were co-expressed (Figure 3A). When ZmFNSI-1, ZmFNSI-2 and ZmS3H were separately transiently co-expressed with Rp1-D21:HA in *N. benthamiana*, ZmFNSI-1 and ZmFNSI-2, but not ZmS3H, suppressed Rp1-D21-mediated HR (Figure 3A). Consistent with our visual observations, ZmFNSI-1, ZmFNSI-2 and HCT1806 significantly reduced ion leakage levels compared to GUS or ZmS3H when co-expressed with Rp1-D21:HA (Figure 3B). Western blot results showed that co-expression of ZmFNSI-1, ZmFNSI-2 and ZmS3H did not have obvious change of the Rp1-D21 expression, compared to co-expression of GUS:EGFP (Figure 3C).

### 2.3. ZmFNSI-1 and ZmFNSI-2 Have No Obvious Suppression Roles on Other Elicitor-Induced HR

To determine whether ZmFNSI-1 and ZmFNSI-2 could suppress HR mediated by other NLR proteins, we co-expressed them with barley MLA10(D502V) and *Arabidopsis* RPM1(D505V), two CNL proteins conferring an autoactive HR when transiently expressed in *N. benthamiana* [39,40]. The results showed that ZmFNSI-1 and ZmFNSI-2 have no obvious suppression roles on either MLA10(D502V)- or RPM1(D505V)-mediated HR (Figure S1A). Similarly, ZmS3H and HCT1806 also have no obvious effects on MLA10(D502V)- or RPM1(D505V)-mediated HR (Figure S1A).

INF1 is a cell death elicitor from *Phytophthora infestans* and Bax is a death-promoting protein of the Bcl-2 family from mouse, both of which can induce cell death when transiently expressed in *N. benthamiana* [41,42]. When co-expressed with Bax or INF1, ZmFNSI-1 and ZmFNSI-2 did not suppress Bax- or INF1-induced cell death, which is similar to the co-expression of ZmS3H or HCT1806 with Bax or INF1, respectively (Figure S1B).

### 2.4. The Enzymatic Activity of ZmFNSI-1 and ZmFNSI-2 Might Not Be Required for Suppressing Rp1-D21-Mediated HR

**Figure 4:**
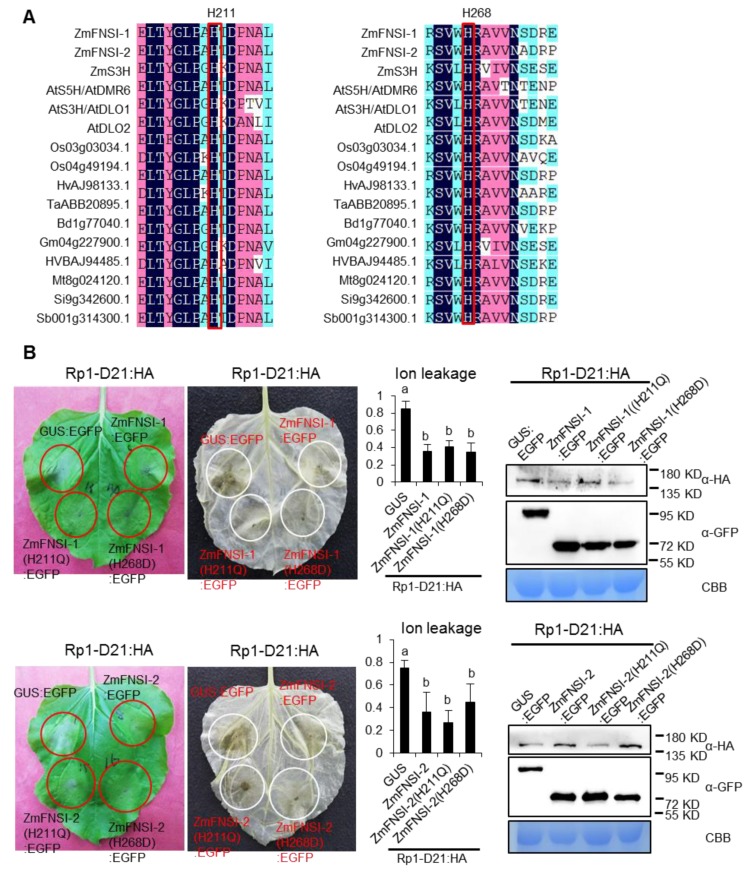
Mutation in the predicted catalytic active site of ZmFNSIs still suppressed Rp1-D21-mediated HR. (**A**) Multiple sequence alignments of FNSIs from different plant species were aligned by DNAMAN software. The conserved His (H) residues at position 211 and 268 of ZmFNSIs are boxed. (**B**) ZmFNSIs and their mutant derivatives were transiently co-expressed with Rp1-D21 into *N. benthamiana*. The representative leaf was photographed at 3 days after inoculation (left), and the same leaf was cleared by ethanol (middle). Ion leakage conductivity (average ± standard error (SE), *n* > 5) was measured at 61 h after co-expression of GUS and ZmFNSI-drivations with Rp1-D21. Significant differences (*p* < 0.05) between samples are indicated by different letters (a–b). Total protein was extracted from agro-infiltrated leaves at 48 hpi. Anti-HA was used to detect the expression of Rp1-D21, and anti-GFP was used to detect the expression of GUS, ZmFNSIs and their mutant derivatives. Equal loading of protein samples was shown by CBB staining of Rubisco.

In *Arabidopsis* AtS5H/AtDMR6, the histidine (H) at positions 212 and 269 is the catalytic residue for binding the ferrous iron atom and is critical for its FNSI enzyme activity [36]. The substitution proteins AtDMR6(H212Q) and AtDMR6(H269D) cannot restore the susceptibility of *Atdmr6* to *H. arabidopsisdis*, indicating that the enzyme activity is required for the function of AtDMR6 [36]. Based on the sequence alignment, we found that the corresponding histidines (H211 and H268 in ZmFNSI-1 and ZmFNSI-2, respectively) are conserved among different plant FNSIs (Figure 4A). To investigate whether the enzymatic activity of ZmFNSI-1 and ZmFNSI-2 is important for inhibiting Rp1-D21-mediated HR, we performed the corresponding mutations in ZmFNSI-1 and ZmFNSI-2 to generate ZmFNSI-1(H211Q), ZmFNSI-2(H211Q), ZmFNSI-1(H268D), and ZmFNSI-2(H268D). When co-expressed with Rp1-D21, all the mutants still suppressed Rp1-D21-mediated HR (Figure 4B). All the HA- and EGFP-tagged proteins were expressed at substantial and broadly comparable levels (Figure 4B). These data suggest that the enzymatic activity of ZmFNSI-1 and ZmFNSI-2 is not required for its function in suppressing Rp1-D21-mediated HR.

### 2.5. ZmFNSI-1 and ZmFNSI-2 Suppress CC_D21_-Mediated HR and Interact with CC_D21_

**Figure 5:**
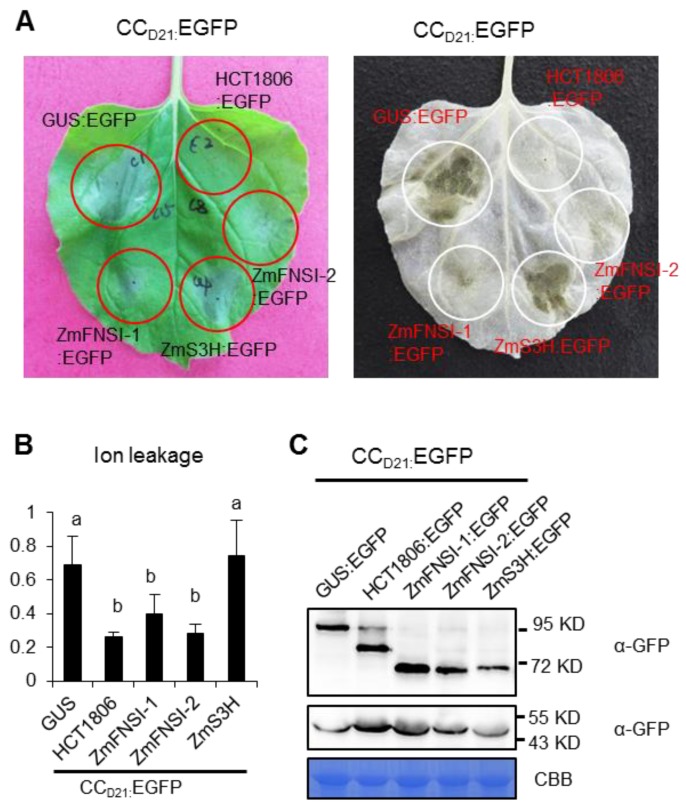
Investigating the function of ZmFNSIs and ZmS3H in CC_D21_ induced HR. (**A**) ZmFNSIs and ZmS3H were transiently co-expressed with CC_D21_ into *N. benthamiana*. The representative leaf was photographed at 3 days after inoculation (left), and the same leaf was cleared by ethanol (right). (**B**) Ion leakage conductivity (average ± SE, *n* > 5) was measured at 61 h after co-expression of GUS, HCT, ZmFNSIs or ZmS3H with CC_D21_. Significant differences (*p* < 0.05) between samples are indicated by different letters (a–b). (**C**) Total protein was extracted from agro-infiltrated leaves at 30 hpi. Anti-GFP was used to detect the expression of GUS, HCT, ZmFNSIs and ZmS3H. Equal loading of protein samples was shown by CBB staining of Rubisco.

The Rp1-D21 protein can be sub-divided into three major domains: an N-terminal coiled-coil (CC) domain termed CC_D21_, a middle NB-ARC (APAF1, certain *R* gene products and CED-4) domain and a C-terminal LRR domain [38]. We have shown previously that CC_D21_, but not NB or LRR, conferred autoactive HR when fused with EGFP and expressed transiently in *N. benthamiana* [38]. To determine whether ZmFNSI-1 and ZmFNSI-2 can suppress CC_D21_-mediated HR, we co-expressed them and CC_D21_ in *N. benthamiana*, and found that ZmFNSI-1 and ZmFNSI-2, but not ZmS3H, suppressed CC_D21_-mediated HR (Figure 5A). Ion leakage analysis further verified the visual observations: co-expression of ZmFNSI-1 and ZmFNSI-2, but not of ZmS3H, with CC_D21_ reduced ion leakage due to CC_D21_-mediated HR (Figure 5B).

**Figure 6:**
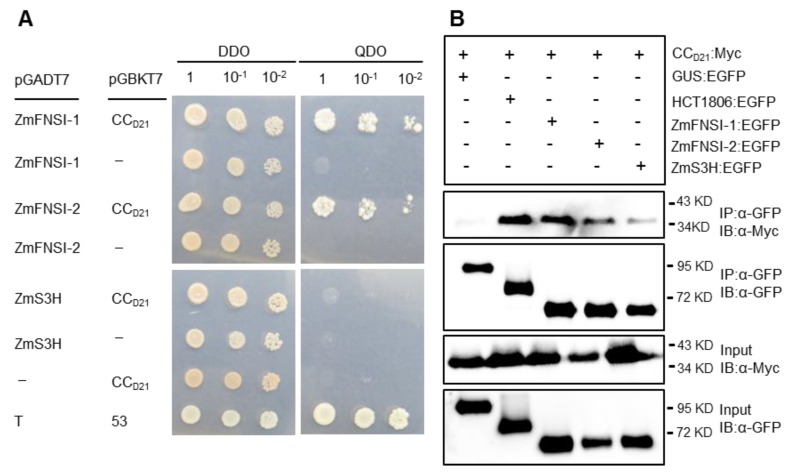
Investigating the interactions between CC_D21_ and ZmFNSIs or ZmS3H. (**A**) Investigating the interactions between CC_D21_ and ZmFNSIs or ZmS3H by yeast two hybridization (Y2H) assay. CC_D21_ was constructed into pGBKT7 and ZmFNSIs and ZmS3H were constructed into pGADT7. SV40 large T-antigen (T) and murine p53 (53) were used as the positive controls. “−” indicated empty vector. DDO: SD-Leu-Trp; QDO: SD-Leu-Trp-Ade-His. (**B**) Investigating the interactions between CC_D21_ and ZmFNSIs or ZmS3H by co-immunoprecipitation (Co-IP) assay. EGFP- and 4×c-Myc-tagged constructs were transiently co-expressed in *N. benthamiana* and samples were collected at 30 hpi for the Co-IP assay. Protein extracts were immunoprecipitated (IP) by anti-GFP (α-GFP) microbeads and detected (immunblotted; IB) by anti-GFP and anti-Myc (α-Myc) antibodies.

We previously showed that HCT1806 interacts with CC_D21_ and suppresses CC_D21_-mediated HR [20]. To investigate whether ZmFNSIs can interact with CC_D21_, we used yeast two hybridization (Y2H) to show that ZmFNSI-1 and ZmFNSI-2 physically associated with CC_D21_ at 72 h after co-expression, while ZmS3H showed weak or no interaction with CC_D21_ (Figure 6A). We further tested the interaction via co-immunoprecipitation (Co-IP) and found that, like HCT1806, ZmFNSI-1 and ZmFNSI-2 interacted strongly with CC_D21_, while ZmS3H interacted weakly with CC_D21_ (Figure 6B), indicating that the interactions between ZmFNSI-1/ ZmFNSI-2 and CC_D21_ are stronger than the interaction between ZmS3H and CC_D21_.

### 2.6. ZmFNSI-1 and ZmFNSI-2 Interact with HCT

As well as HCT1806, we have shown that CCoAOMT2 can also suppress Rp1-D21-mediated HR and can physically interact with CC_D21_ [20,21]. To investigate whether ZmFNSI-1 or ZmFNSI-2 can interact with HCT or CCoAOMT2, we performed a Y2H assay. The results showed that both ZmFNSI-1 and ZmFNSI-2 interacted with HCT, but not with CCoAOMT2 (Figure S2). Interestingly, ZmS3H also interacted with HCT and interacted weakly with CCoAOMT2 (Figure S2).

### 2.7. ZmFNSI-1 and ZmFNSI-2 Form Stronger Homomers than ZmS3H

We further used yeast two hybridization (Y2H) to test whether ZmFNSI-1, ZmFNSI-2 and ZmS3H can form homomers. As shown in Figure S3, ZmFNSI-1 and ZmFNSI-2 self-associated at 52 h after co-expression, while ZmS3H showed weak self-association until 81 h after co-expression.

### 2.8. ZmFNSI-1 and ZmFNSI-2 do not Change the Subcellular Localization of CC_D21_

To investigate where ZmFNSI-1, ZmFNSI-2 and ZmS3H are localized, we performed subcellular localization experiments by transient expression in transgenic *N. benthamiana* lines harboring the nuclear marker H2B:RFP [43]. When fused with a C-terminal EGFP, ZmFNSI-1, ZmFNSI-2 and ZmS3H mainly distributed in cytoplasm and nucleus (Figure S4).

Previously we showed that Rp1-D21 and CC_D21_ localized to both the cytoplasm and nucleus, and that nucleo-cytoplasm trafficking was important for HR induction [44]. To determine whether the different suppression effects between ZmFNSI-1, ZmFNSI-2 and ZmS3H were due to their different effects on changing the subcellular localization of CC_D21_, we co-expressed ZmFNSI-1, ZmFNSI-2 and ZmS3H with CC_D21_:RFP in *N. benthamiana*. When CC_D21_:RFP was co-expressed with GUS:EGFP, it mainly localized in cytoplasm and nucleus, which is consistent with previous results [44]. Similarly, when co-expressed with ZmFNSI-1, ZmFNSI-2 and ZmS3H, CC_D21_:RFP also mainly localized in the cytoplasm and nucleus (Figure S5). The results suggest that the suppression effect of ZmFNSI-1 and ZmFNSI-2 on CC_D21_-mediated HR was not due to change the subcellular localization of CC_D21_.

## 3. Discussion

### 3.1. ZmFNSI Functions in Plant Defense Response

FNSI is involved in the biosynthesis of secondary metabolites [29,30]. ZmFNSI-1 was reported to have FNSI activity which can catalyze the conversion of flavanones into flavones [28]. AtDMR6, the *Arabidopsis* homologous enzyme to ZmFNSI, also has FNSI activity [28]. The loss-of-function mutant *Atdmr6* confers resistance to multiple pathogens [29,36], and overexpression of ZmFNSI-1 in *drm6-1* mutant plants results in reduced resistance to *P. syringae* [28], indicating that the function of FNSI/DMR6 in *Arabidopsis* and maize is conserved in plant defense response. CRISPR-Cas9 mediated knockout of the tomato ortholog *SlDMR6-1* confers resistance against different pathogens, including *P. syringae*, *Xanthomonas* spp. and *P. capsici* [45]. These results indicate that FNSI/DMR6 from different plant species acts as a negative regulator in the plant defense response.

We had previously found that many genes predicted to be involved in secondary metabolism were induced by Rp1-D21, including the phenylpropanoid pathway [20,21]. The CCoAOMT2 and HCT1806 proteins were predicted to catalyze consecutive steps in the lignin biosynthesis pathway and have been implicated to play important roles in Rp1-D21-mediated HR [21]. CCoAOMT2 has also been shown to confer resistance to southern leaf blight and gray leaf spot in maize [46]. From previous transcriptional data, *ZmFNSI-1* and *HCT1806* were highly induced in maize lines inoculated by several fungal pathogens which cause ear rot, including *Fusarium graminearum*, *Fusarium verticillioides* and *Aspergillus flavus* [27,47,48,49,50]. *ZmFNSI-1*, *ZmS3H*, *CCoAOMT2* and *HCT1806* are also upregulated in maize lines expressing WtsE from *Pnss* [51], suggesting these genes are also involved in maize disease resistance. Several examples have shown that negative regulators of plant immunity are induced during the defense response to control and prevent the over-activation of the defense response, e.g., Nudix hydrolase-encoding NUDT7 [52], HCT1806 and CCoAOMT2 [20,21]. Here, we found that the transcriptional levels of the genes encoding ZmFNSI-1, ZmFNSI-2, HCT1806 and CCoAOMT2 were significantly induced in the *Rp1-D21* mutant. ZmFNSI-1 and ZmFNSI-2 acted as negative regulators to suppress Rp1-D21-mediated HR and they interacted with the CC signaling domain of Rp1-D21, suggesting that ZmFNSI/ZmS5H regulates Rp1-D21-mediated HR, likely through physical interaction. We speculate that the increased levels of ZmFNSI-1 and ZmFNSI-2 can serve as a reservoir to inhibit the further activation of Rp1 protein and the spread of HR.

Tricin, a flavone constituted by modified apigenins, is a structural monomer of lignin in maize [53]. Falcone Ferreyra et al. (2015) suggested that ZmFNSI-1 might have a role in lignin biosynthesis. In this study, we performed Y2H assays and found that ZmFNSI-1 and ZmFNSI-2 interacted with HCT, the key enzyme in the lignin biosynthesis pathway. This interaction, together with the interaction between HCT and CCoAOMT2 [21], may reflect the formation of a metabolon, a complex formed between sequential enzymes of a metabolic pathway believed to facilitate metabolite processing [54]. Components of the phenylpropanoid pathway have been shown to associate with each other in poplar and other species [55].

### 3.2. FNSI Enzyme Activity Is not Required for HR and ZmFNSIs Are not General Cell Death Suppressors

FNS1 belongs to the 2OGD superfamily members. In *Arabidopsis*, AtDMR6 acts as a negative regulator in plant immunity. Expression of AtDMR6(H212Q) and AtDMR6(H269D), the substitution proteins in the histidine (H) residues required for the putative catalytic activity of AtDMR6, fail to restore the susceptibility of *Atdmr6* to *H. arabidopsidis*, suggesting that the oxygenase enzymatic activity of AtDMR6 is required for its function in disease resistance [36]. For the maize ZmFNS1-1 and ZmFNS1-2, however, the corresponding mutation in ZmS5H-1(H211Q), ZmS5H-2(H211Q), ZmS5H-1(H268D), and ZmS5H-2(H268D) still suppress Rp1-D21-mediated HR (Figure 4), suggesting that the enzyme activity is not required for the suppression effect. It is also possible that the substitution mutants retained low enzymatic activity which is sufficient to suppress HR. To exclude the possibility, the enzymatic activity for these mutants will be further determined. As mentioned above, *ZmFNSI* is induced by multiple pathogens. *ZmFNSI-1* is located in a quantitative trait loci (QTL) conferring resistance to southern leaf blight [56], and it is also located at approximately 380 kb upstream of a SNP (s-ingle nucleotide polymorphism) which is associated with multiple disease resistance [57]. These results suggest that ZmFNSI might be also involved in maize disease resistance. It is possible that the differential enzyme activity requirement of FNS1/DMR6 in disease resistance and HR might be uncoupled. AtFNS1/AtDMR6 in *Arabidopsis* also has S5H activity [36]; it will be interesting to investigate whether ZmFNSI-1 and ZmFNSI-2 have S5H activity and whether the S5H activity is involved in Rp1-D21-mediated HR.

ZmFNSI-1 and ZmFNSI-2 suppressed Rp1-D21- and CC_D21_-mediated HR, however, they did not substantially suppress the HR phenotype caused by the other two CNL autoactive mutants RPM1(D505V) and MLA10(D502V), and they have no obvious effect on Bax- and INF1-mediated cell death. These data suggested that ZmFNSI-1 and ZmFNSI-2 might not be general HR suppressors.

### 3.3. FNSI/S5H and S3H have Overlapping and Different Roles in Plant Defense Response

Plant resistance to biotrophic/hemibiotrophic pathogens is largely controlled by SA-mediated signaling pathways [58]. The compounds 2,5-DHBA and 2,3-DHBA are two major catabolic products of SA and they are catalyzed by hydroxylating SA via S5H and S3H, respectively [35,37]. In *Arabidopsis*, AtDMR6/AtS5H was reported to have high S5H activity and low FNSI activity [35]. Therefore, the enhanced disease resistance of *Atdmr6* is most likely caused by increased total SA levels [35]. After *Pst* DC3000 treatment, the transcript levels of *AtDMR6/AtS5H* and *AtS3H* are induced, and the accumulation of 2,5-DHBA and 2,3-DHBA is enhanced, implying that SA hydroxylation plays a role in the detoxification of excessive SA [35]. *AtDMR6/AtS5H* is more sensitive to SA treatment and pathogen induction than *AtS3H* and the *Ats5h* mutant exhibited much stronger pathogen resistance than the *Ats3h* mutant [29,35,36]. The SA levels are higher in the *Atdmr6* mutant compared to wild type, and there is no significant different between the *dlo1* mutant and wild type. However, in the *dmr6dlo1* double mutant, the SA levels are approximately 20 times higher than that in the *Atdmr6* mutant [36]. Therefore, *AtDMR6/AtS5H* and *AtS3H* have overlapping and distinct functions as negative regulators in innate immunity. Here, we found that the fold change of the transcript levels of *ZmFNSI-1* and *ZmFNSI-2* is higher than that of *ZmS3H* in the *Rp1-D21* mutant compared to the corresponding wild type backgrounds (Table 1), and ZmFNSI-1 and ZmFNSI-2, but not ZmS3H, suppress Rp1-D21- and CC_D21_-mediated HR, indicating the different roles of ZmFNSI and ZmS3H in the maize defense response. We also found ZmFNSI-1 and ZmFNSI-2 formed stronger homomers than ZmS3H (Figure S3), however, it needs to be further investigated whether the formation of homomers is related with the different roles of ZmFNSIs and ZmS3H in Rp1-D21-mediated HR.

### 3.4. ZmFNSI Regulates Rp1-D21-mediated HR possibly through Forming a Protein Complex

Plant NLR genes are usually expressed at low levels in living cells. The over-accumulation of NLRs can lead to autoimmune responses, causing dwarfism and an autoactive HR phenotype [59]. To reduce the pleiotropic effects to plant growth and development, NLR proteins are usually kept in an inactive state by a delicate combination of intra-molecular and inter-molecular interactions. Several different categories of host proteins (including transcription factors, kinases, E3 ligases, etc.) physically interact with NLRs to modulate plant innate immunity and some NLRs can interact with different host proteins targeted by different pathogen effectors [60]. For instance, in *Arabidopsis*, the NLR protein ZAR1 (HOPZ-ACTIVATED RESISTANCE1) interacts with several kinases or pseudokinases to recognize effectors secreted from different pathogens [61,62,63]. Recent cryo-electron microscopy studies have characterized the structures of ZAR1 complexes in different states; the inactive ZAR1-RKS1, the intermediate ZAR1-RKS1-PBL2^UMP^ and the activated ZAR1-RKS1-PBL2^UMP^ complexes [64,65]. A wheel-like pentameric ZAR1 “resistosome” is formed in the activated state that is believed to directly induce the defense response, including HR [65].

Rp1-D confers resistance to common rust caused by *P. sorghi* [66]. However, the cognate effector of Rp1-D from *P. sorghi* is still unknown. In our previous studies, we derived models to elucidate the transition between the resting and activation states of Rp1 proteins [20,21,38]. We proposed that in the resting state, Rp1 proteins are maintained by intramolecular interactions, some of which we defined, and also by interaction with other host proteins which acted as negative regulators, including HCT and CCoAOMT2. We now propose that ZmFNSI-1 and ZmFNSI-2 are also involved in the negative regulation of Rp1-D. In the activated state, we speculate that unknown effectors from a pathogen can target one of the components from HCT, CCoAOMT2 or ZmFNSIs and disrupt their association with Rp1 proteins, leading to the activation of Rp1. We have also previously shown that Rp1 proteins can form homomers [38]. By analogy with the ZAR1 resistosome, we speculate that Rp1-D, ZmFNSIs, HCT or CCoAOMT2 might also form a ‘Rp1-D resistosome’ to function in the maize defense response.

In summary, we have demonstrated that ZmFNSI-1 and ZmFNSI-2, two homologs of enzymes which catalyze key steps in the flavone biosynthesis and SA catabolism pathway, interact with the CC domain of Rp1-D21 to regulate Rp1-D21 -mediated HR. ZmFNSI-1 and ZmFNSI-2 also interact with HCT. To our knowledge, this is the first evidence that the homologs of enzymes in the flavonoid and lignin biosynthesis pathways may function together to regulate the NLR-mediated defense response. Though substantial data were derived from agrobacteria-mediated transient expression in *N. benthamiana*, we believe that it can largely reflect the situation in maize for the following reasons. Firstly, this system has been widely employed for exploring the HR phenotype mediated by NLRs from diverse plant species [39,67,68,69,70]. Secondly, we have used this system to verify the suppression roles of HCT1806, HCT4918 and CCoAOMT2 in Rp1-D21-mediated HR [20,21]. Thirdly, we have also used this system to verify the HR conferred by 12 EMS maize mutants derived from Rp1-D21 and it can recapitulate the HR phenotype in maize [38]. With the maize genetic materials available in the future, we hope to further verify the results in this study.

## 4. Materials and Methods

### 4.1. Plant Materials and Growth Condition

Maize (*Zea mays*) line B73 was grown at 24 °C with 12 h light/12 h dark conditions and used for isolating *ZmFNSI-1*, *ZmFNSI-2* and *ZmS3H*. Wild type *Nicotiana benthamiana* and the transgenic *N. benthamiana* harboring histone 2B (H2B)-TaqRFP were grown at 23 °C with a cycle of 16 h light and 8 h dark.

### 4.2. Sequence Alignment and Phylogenetic Analysis

For phylogenetic analysis, the protein sequences from the FNSI and S3H families were aligned using Clustal X v2.1 [71]. Based on this alignment, a neighbor-joining tree was constructed using MEGA 6.0 software with 1000 bootstrap replicates [72].

### 4.3. Plasmid Construction

GUS:EGFP, HCT1806:EGFP, CC_D21_:EGFP, CC_D21_:Myc and Rp1-D21:HA were generated previously [20,38]. The cDNA of *ZmFNSI-1*, *ZmFNSI-2* and *ZmS3H* were isolated from B73 inbred line using primers listed in Table S1 and cloned into pENTR directional TOPO cloning vector (D-TOPO, Invitrogen, CA, USA). After sequencing, they were constructed into pSITEII-N1-EGFP vector [43] by LR reactions.

### 4.4. Agrobacterium tumefaciens-Mediated Transient Expression

*A. tumefaciens* strain GV3101 (pMP90) transformed with binary vector constructs was grown at 28 °C overnight in 8 mL of L-broth medium supplemented with appropriate antibiotics. The detailed procedures were performed according to our previous study [38]. Unless otherwise indicated, all the experiments were repeated three times with similar results.

### 4.5. Protein Analysis

For protein expression analysis, three leaf discs (1.2 cm in diameter) infiltrated by agrobacteria were collected at 30 h post inoculation (hpi). The samples were ground in liquid nitrogen, and total protein was extracted in 150 µL extraction buffer (20 mM Tris·HCl (pH 8.0), 150 mM NaCl, 1 mM EDTA (pH 8.0), 1% Triton X-100, 0.1% SDS, 10 mM DTT, 40 µM MG132, and 1× plant protein protease inhibitor mixture (Sigma-Aldrich, Louis, MS, USA)). For the Co-IP assay, Myc-, EGFP-, and 3×HA-tagged constructs were transiently co-expressed into *N. benthamiana*. Leaf samples were collected at 30 hpi, and proteins were extracted by grinding 0.6 g of leaf samples in 1.8 mL extraction buffer (50 mM HEPES (pH 7.5), 50 mM NaCl, 10 mM EDTA (pH 8.0), 0.5% Triton 100, 4 mM DTT, 40 µM MG132, and 1× plant protein protease inhibitor mixture (Sigma-Aldrich, Louis, MS, USA). The kit containing anti-GFP microbeads (Cat# 130-091-125, Miltenyi Biotec, Bergisch Gladbach, Germany) was used for the Co-IP assay. The detailed procedures for the Co-IP assay were performed according to our previous study [38] with some modifications. HA detection was performed using a 1:6000 dilution of primary rabbit polyclonal anti-HA (Cat# H6908, Sigma-Aldrich, Louis, MS, USA), followed by hybridization with a 1:5000 dilution of anti-rabbit-HRP second antibody (Cat# A00098, GenScript, Nanjing, China). Myc detection was performed using a 1:6000 dilution of primary mouse monoclonal anti-Myc (Cat# A00704S, GenScript, Nanjing, China), followed by hybridization with a 1:5000 dilution of anti-mouse-HRP second antibody (Cat# A00160, GenScript, Nanjing, China). GFP detection was performed using a 1:6000 dilution of primary mouse polyclonal anti-GFP (Cat# A01704S, GenScript, Nanjing, China), followed by hybridization with a 1:5000 dilution of anti-mouse-HRP second antibody. The HRP signal was detected by an ECL substrate kit (Supersignal west femto chemiluminescent substrate, Thermo Scientific, Waltham, MA, USA).

### 4.6. Y2H

ZmFNSI-1, ZmFNSI-2 and ZmS3H were constructed into the pGADT7 (AD) cloning vector (Clontech, Mountain View, CA, USA) by LR reactions, and CC_D21_ were constructed into the pGBKT7 (BD) vector (Clontech, Mountain View, CA, USA). AD- and BD-derived constructs were co-transformed into competent cells of the yeast strain Y2HGold. The Y2H interaction assay was performed according to the protocol provided by the manufacturer (Clontech, Mountain View, CA, USA).

### 4.7. Confocal Microscopy

Confocal microscopy observation was performed according to our previous study [44] with some modifications. After agrobacteria infiltration, the abaxial sides of *N. benthaminana* leaves were used for observation at 48 hpi by a confocal microscope (LSM 700, Carl Zeiss, Jena, Germany) with a PMT detector. The representative areas were photographed under a 20× objective. EGFP fluorescence was excited at 488 nm and observed between 495 and 550 nm. RFP was excited at 561 nm and observed between 580 and 675 nm.

### 4.8. Accession Number

Sequence data from this article can be found in the EMBL/GenBank data libraries under the following accession numbers. Nucleotide sequence and protein sequence of *Rp1-D21* is KF951062 and AIW65617, respectively. RNA-seq data: SRP060286.

## Figures and Tables

**Table 1:** The fold change (FC) of the transcript levels of maize genes in *Rp1-D21* compared to the corresponding wild type and the effect of the genes on the Rp1-D21-mediated hypersensitive response (HR).

Gene Name	Accession Number	Chromosomal Position	FC in B73 × H95 Isogenic Lines	FC in Mo17 × H95 Isogenic Lines	Rp1-D21-Mediated HR Phenotype
ZmFNSI-1	Zm00001d029744	Chr1: 85,068,069..85,072,051	17.23	55.62	Suppressed
ZmFNSI-2	Zm00001d027423	Chr1: 4,909,652..4,914,685	235.02	28.01	Suppressed
ZmS3H	Zm00001d002564	Chr2: 15,538,202..15,540,469	8.56	4.18	No effect
HCT1806	Zm00001d027946	Chr1: 18,086,164..18,087,981	292.03	183.11	Suppressed
HCT4918	Zm00001d027948	Chr1: 18,147,157..18,148,937	1115	568.5	Suppressed
CCoAOMT2	Zm00001d045206	Chr9: 16,074,658..16,083,126	2.07	1.70	Suppressed
Peptidoglycan related genes	Zm00001d043988	Chr3: 215,679,092..215,679,979	250.59	69.97	No effect
	Zm00001d027325	Chr1: 2,974,258..2,976,935	2.36	3.06	No effect
	Zm00001d053695	Chr4: 239,190,056..239,192,283	22.92	12.59	No effect
Wall associated kinase	Zm00001d003019	Chr2: 29,627,526..29,635,338	5.82	4.84	Autoactive HR
	Zm00001d003021	Chr2: 29,666,263..29,670,199	6.08	4.61	Autoactive HR
Auxin-responsive genes	Zm00001d033460	Chr1: 263,312,679..263,313,005	24.76	177.41	No effect
	Zm00001d031666	Chr1: 195785845-195789120	18.10	21.15	No effect
	Zm00001d028167	Chr1: 25,047,706..25,050,015	41.12	84.14	No effect
MTHFR2	Zm00001d034602	Chr1: 297,605,177..297,611,407	4.33	4.76	No effect
Calmodulin binding proteins	Zm00001d023843	Chr10: 24,286,776..24,290,769	157.85	85.47	Autoactive HR
	Zm00001d052525	Chr4: 192,080,883..192,084,967	4.55	4.41	No effect
	Zm00001d004916	Chr2: 148,560,206..148,564,300	7.69	2.17	No effect
EF hand family	Zm00001d043258	Chr3: 193,663,756..193,664,037	10.66	45.62	Partially suppressed
UDP-glycosyltransferase	Zm00001d014126	Chr5: 33,393,733..33,395,460	5.45	1.91	No effect
Cytochrome B5 isoforms	Zm00001d017425	Chr5: 195,722,803..195,723,646	123.41	163.02	Enhanced
	Zm00001d011081	Chr8: 138,418,132..138,420,302	2.27	2.74	Enhanced

## Supplementary Materials

Supplementary materials can be found at https://www.mdpi.com/1422-0067/21/7/2529/s1. Figure S1. Investigating the function of ZmFNSIs and ZmS3H in other elicitor-mediated HR. ZmFNSIs have no obvious suppressive roles on MLA(D502V)-, RPM1(D505V)-, INF1- or Bax-mediated HR. GUS, HCT1806, ZmFNSIs and ZmS3H were transiently co-expressed with RPM1(D505V), MLA(D502V), INF1 or Bax into *N. benthamiana*. The representative leaf was photographed at 3 days after inoculation. Figure S2. Investigating the interactions between HCT1806 or CCoAOMT2 and ZmFNSIs or ZmS3H. HCT1806 and CCoAOMT2 were constructed into pGBKT7 and ZmFNSIs and ZmS3H were constructed into pGADT7. T + 53 was used as the positive control. “−” indicated empty vector. Figure S3. Investigating the self-association of ZmFNSIs and ZmS3H. ZmFNSIs and ZmS3H were constructed into pGADT7 and pGBKT7 vectors. T + 53 was used as the positive control. “−” indicated empty vector. Figure S4. The subcellular localization of ZmFNSIs and ZmS3H. ZmFNSIs and ZmS3H were fused with C-terminal EGFP, and infiltrated into *N. benthamiana* transformed with nuclear marker H2B-TaqRFP. Confocal images were taken at 48 hpi. The position of the nucleus was labeled by arrows. The scale bar represents 50 µm. The experiment was repeated three times with the same results. Figure S5. ZmFNSIs and ZmS3H did not change the subcellular localization of CC_D21_. ZmFNSIs:EGFP or ZmS3H:EGFP were co-infiltrated with CC_D21_:TaqRFP into *N. benthamiana*, and confocal images were taken at 48 hpi. The positions of the nuclei were labeled by arrows. The scale bar represents 50 µm. The experiment was repeated three times with the same results. Table S1. The primers used in this study.

## References

[1] Jones J.D., Dangl J.L. (2006). The plant immune system. Nature.

[2] Wang W., Feng B., Zhou J.M., Tang D. (2020). Plant immune signaling: Advancing on two frontiers. J. Integr. Plant Biol..

[3] Bent A.F., Mackey D. (2007). Elicitors, effectors, and R genes: The new paradigm and a lifetime supply of questions. Annu. Rev. Phytopathol..

[4] Balint-Kurti P. (2019). The plant hypersensitive response: Concepts, control and consequences. Mol. Plant Pathol..

[5] Cui H., Tsuda K., Parker J.E. (2015). Effector-triggered immunity: From pathogen perception to robust defense. Annu. Rev. Plant Biol..

[6] Mur L.A., Kenton P., Lloyd A.J., Ougham H., Prats E. (2008). The hypersensitive response; the centenary is upon us but how much do we know?. J. Exp. Bot..

[7] Kourelis J., van der Hoorn R.A.L. (2018). Defended to the nines: 25 Years of resistance gene cloning identifies nine mechanisms for R protein function. Plant Cell.

[8] Dangl J.L., Jones J.D. (2001). Plant pathogens and integrated defence responses to infection. Nature.

[9] Ellis J., Dodds P., Pryor T. (2000). Structure, function and evolution of plant disease resistance genes. Curr. Opin. Plant Biol..

[10] Monteiro F., Nishimura M.T. (2018). Structural, functional, and genomic diversity of plant NLR proteins: An evolved resource for rational engineering of plant immunity. Annu. Rev. Phytopathol..

[11] Hulbert S.H. (1997). Structure and evolution of the rp1 complex conferring rust resistance in maize. Annu. Rev. Phytopathol..

[12] Sudupak M.A., Bennetzen J.L., Hulbert S.H. (1993). Unequal exchange and meiotic instability of disease-resistance genes in the Rp1 region of maize. Genetics.

[13] Sun Q., Collins N.C., Ayliffe M., Smith S.M., Drake J., Pryor T., Hulbert S.H. (2001). Recombination between paralogues at the Rp1 rust resistance locus in maize. Genetics.

[14] Smith S.M., Steinau M., Trick H.N., Hulbert S.H. (2010). Recombinant Rp1 genes confer necrotic or nonspecific resistance phenotypes. Mol. Genet. Genom..

[15] Negeri A., Wang G.F., Benavente L., Kibiti C.M., Chaikam V., Johal G., Balint-Kurti P. (2013). Characterization of temperature and light effects on the defense response phenotypes associated with the maize Rp1-D21 autoactive resistance gene. BMC Plant Biol..

[16] Chintamanani S., Hulbert S.H., Johal G.S., Balint-Kurti P.J. (2010). Identification of a maize locus that modulates the hypersensitive defense response, using mutant-assisted gene identification and characterization. Genetics.

[17] Chaikam V., Negeri A., Dhawan R., Puchaka B., Ji J., Chintamanani S., Gachomo E.W., Zillmer A., Doran T., Weil C. (2011). Use of Mutant-Assisted Gene Identification and Characterization (MAGIC) to identify novel genetic loci that modify the maize hypersensitive response. Theor. Appl. Genet..

[18] Olukolu B.A., Negeri A., Dhawan R., Venkata B.P., Sharma P., Garg A., Gachomo E., Marla S., Chu K., Hasan A. (2013). A connected set of genes associated with programmed cell death implicated in controlling the hypersensitive response in maize. Genetics.

[19] Olukolu B.A., Wang G.F., Vontimitta V., Venkata B.P., Marla S., Ji J., Gachomo E., Chu K., Negeri A., Benson J. (2014). A genome-wide association study of the maize hypersensitive defense response identifies genes that cluster in related pathways. PLoS Genet..

[20] Wang G.F., He Y., Strauch R., Olukolu B.A., Nielsen D., Li X., Balint-Kurti P.J. (2015). Maize homologs of hydroxycinnamoyltransferase, a key enzyme in lignin biosynthesis, bind the nucleotide binding leucine-rich repeat Rp1 proteins to modulate the defense response. Plant Physiol..

[21] Wang G.F., Balint-Kurti P.J. (2016). Maize homologs of CCoAOMT and HCT, two key enzymes in lignin biosynthesis, form complexes with the NLR Rp1 protein to modulate the defense response. Plant Physiol..

[22] Padmavati M., Reddy A.R. (1999). Flavonoid biosynthetic pathway and cereal defence response: An emerging trend in crop biotechnology. J. Plant Biochem. Biot..

[23] Fofana B., Benhamou N., McNally D.J., Labbe C., Seguin A., Belanger R.R. (2005). Suppression of induced resistance in cucumber through disruption of the flavonoid pathway. Phytopathology.

[24] Gill U.S., Uppalapati S.R., Gallego-Giraldo L., Ishiga Y., Dixon R.A., Mysore K.S. (2018). Metabolic flux towards the (iso)flavonoid pathway in lignin modified alfalfa lines induces resistance against *Fusarium oxysporum* f. sp *medicaginis*. Plant Cell Environ..

[25] George V.C., Dellaire G., Rupasinghe H.P.V. (2017). Plant flavonoids in cancer chemoprevention: Role in genome stability. J. Nutr. Biochem..

[26] Peters N.K., Frost J.W., Long S.R. (1986). A plant flavone, luteolin, induces expression of Rhizobium meliloti nodulation genes. Science.

[27] Lanubile A., Ferrarini A., Maschietto V., Delledonne M., Marocco A., Bellin D. (2014). Functional genomic analysis of constitutive and inducible defense responses to Fusarium verticillioides infection in maize genotypes with contrasting ear rot resistance. BMC Genom..

[28] Falcone Ferreyra M.L., Emiliani J., Rodriguez E.J., Campos-Bermudez V.A., Grotewold E., Casati P. (2015). The identification of maize and Arabidopsis type I FLAVONE SYNTHASEs links flavones with hormones and biotic interactions. Plant Physiol..

[29] van Damme M., Huibers R.P., Elberse J., Van den Ackerveken G. (2008). Arabidopsis DMR6 encodes a putative 2OG-Fe(II) oxygenase that is defense-associated but required for susceptibility to downy mildew. Plant J..

[30] Kawai Y., Ono E., Mizutani M. (2014). Evolution and diversity of the 2-oxoglutarate-dependent dioxygenase superfamily in plants. Plant J..

[31] Liu Q., Liu H., Gong Y., Tao Y., Jiang L., Zuo W., Yang Q., Ye J., Lai J., Wu J. (2017). An atypical thioredoxin imparts early resistance to sugarcane mosaic virus in maize. Mol. Plant.

[32] Keskiaho K., Hieta R., Sormunen R., Myllyharju J. (2007). *Chlamydomonas reinhardtii* has multiple prolyl 4-hydroxylases, one of which is essential for proper cell wall assembly. Plant Cell.

[33] Kim J.H., Cheon Y.M., Kim R.G., Ahn J.H. (2008). Analysis of flavonoids and characterization of the OsFNS gene involved in flavone biosynthesis in rice. J. Plant Biol..

[34] Lee Y.J., Kim J.H., Kim B.G., Lim Y., Ahn J.H. (2008). Characterization of flavone synthase I from rice. BMB Rep..

[35] Zhang Y., Zhao L., Zhao J., Li Y., Wang J., Guo R., Gan S., Liu C.J., Zhang K. (2017). S5H/DMR6 encodes a salicylic acid 5-hydroxylase that fine-tunes salicylic acid homeostasis. Plant Physiol..

[36] Zeilmaker T., Ludwig N.R., Elberse J., Seidl M.F., Berke L., Van Doorn A., Schuurink R.C., Snel B., Van den Ackerveken G. (2015). DOWNY MILDEW RESISTANT 6 and DMR6-LIKE OXYGENASE 1 are partially redundant but distinct suppressors of immunity in Arabidopsis. Plant J..

[37] Zhang K., Halitschke R., Yin C., Liu C.J., Gan S.S. (2013). Salicylic acid 3-hydroxylase regulates Arabidopsis leaf longevity by mediating salicylic acid catabolism. Proc. Natl. Acad. Sci. USA.

[38] Wang G.F., Ji J., Ei-Kasmi F., Dangl J.L., Johal G., Balint-Kurti P.J. (2015). Molecular and functional analyses of a maize autoactive NB-LRR protein identify precise structural requirements for activity. PLoS Pathog..

[39] Bai S., Liu J., Chang C., Zhang L., Maekawa T., Wang Q., Xiao W., Liu Y., Chai J., Takken F.L. (2012). Structure-function analysis of barley NLR immune receptor MLA10 reveals its cell compartment specific activity in cell death and disease resistance. PLoS Pathog..

[40] Gao Z., Chung E.H., Eitas T.K., Dangl J.L. (2011). Plant intracellular innate immune receptor Resistance to *Pseudomonas syringae* pv. *maculicola* 1 (RPM1) is activated at, and functions on, the plasma membrane. Proc. Natl. Acad. Sci. USA.

[41] Kamoun S., van West P., Vleeshouwers V.G., de Groot K.E., Govers F. (1998). Resistance of *Nicotiana benthamiana* to *Phytophthora infestans* is mediated by the recognition of the elicitor protein INF1. Plant Cell.

[42] Lacomme C., Santa Cruz S. (1999). Bax-induced cell death in tobacco is similar to the hypersensitive response. Proc. Natl. Acad. Sci. USA.

[43] Martin K., Kopperud K., Chakrabarty R., Banerjee R., Brooks R., Goodin M.M. (2009). Transient expression in *Nicotiana benthamiana* fluorescent marker lines provides enhanced definition of protein localization, movement and interactions in planta. Plant J..

[44] Wang G.F., Balint-Kurti P.J. (2015). Cytoplasmic and nuclear localizations are important for the hypersensitive response conferred by maize autoactive Rp1-D21 protein. Mol. Plant Microbe Interact..

[45] Thomazella D.P.D.T., Brail Q., Dahlbeck D., Staskawicz B. (2016). CRISPR-Cas9 mediated mutagenesis of a DMR6 ortholog in tomato confers broad-spectrum disease resistance. bioRxiv.

[46] Yang Q., He Y., Kabahuma M., Chaya T., Kelly A., Borrego E., Bian Y., El Kasmi F., Yang L., Teixeira P. (2017). A gene encoding maize caffeoyl-CoA O-methyltransferase confers quantitative resistance to multiple pathogens. Nat. Genet..

[47] Liu Y., Guo Y., Ma C., Zhang D., Wang C., Yang Q. (2016). Transcriptome analysis of maize resistance to Fusarium graminearum. BMC Genom..

[48] Kebede A.Z., Johnston A., Schneiderman D., Bosnich W., Harris L.J. (2018). Transcriptome profiling of two maize inbreds with distinct responses to Gibberella ear rot disease to identify candidate resistance genes. BMC Genom..

[49] Musungu B.M., Bhatnagar D., Brown R.L., Payne G.A., OBrian G., Fakhoury A.M., Geisler M. (2016). A network approach of gene co-expression in the *Zea mays*/*Aspergillus flavus* pathosystem to map host/pathogen interaction pathways. Front. Genet..

[50] Shu X., Livingston D.P., Woloshuk C.P., Payne G.A. (2017). Comparative histological and transcriptional analysis of maize kernels infected with *Aspergillus flavus* and *Fusarium verticillioides*. Front. Plant Sci..

[51] Asselin J.E., Lin J., Perez-Quintero A.L., Gentzel I., Majerczak D., Opiyo S.O., Zhao W., Paek S.M., Kim M.G., Coplin D.L. (2015). Perturbation of maize phenylpropanoid metabolism by an AvrE family type III effector from *Pantoea stewartii*. Plant Physiol..

[52] Ge X., Li G.J., Wang S.B., Zhu H., Zhu T., Wang X., Xia Y. (2007). AtNUDT7, a negative regulator of basal immunity in Arabidopsis, modulates two distinct defense response pathways and is involved in maintaining redox homeostasis. Plant Physiol..

[53] Lan W., Lu F., Regner M., Zhu Y., Rencoret J., Ralph S.A., Zakai U.I., Morreel K., Boerjan W., Ralph J. (2015). Tricin, a flavonoid monomer in monocot lignification. Plant Physiol..

[54] Jorgensen K., Rasmussen A.V., Morant M., Nielsen A.H., Bjarnholt N., Zagrobelny M., Bak S., Moller B.L. (2005). Metabolon formation and metabolic channeling in the biosynthesis of plant natural products. Curr. Opin. Plant Biol..

[55] Wang J.P., Liu B., Sun Y., Chiang V.L., Sederoff R.R. (2019). Enzyme-enzyme interactions in monolignol biosynthesis. Front. Plant Sci..

[56] Balint-Kurti P.J., Zwonitzer J.C., Wisser R.J., Carson M.L., Oropeza-Rosas M.A., Holland J.B., Szalma S.J. (2007). Precise mapping of quantitative trait loci for resistance to southern leaf blight, caused by *Cochliobolus heterostrophus* race O, and flowering time using advanced intercross maize lines. Genetics.

[57] Lopez-Zuniga L.O., Wolters P., Davis S., Weldekidan T., Kolkman J.M., Nelson R., Hooda K.S., Rucker E., Thomason W., Wisser R. (2019). Using maize chromosome segment substitution line populations for the identification of loci associated with multiple disease resistance. G3 Genes Genomes Genet..

[58] Vlot A.C., Dempsey D.A., Klessig D.F. (2009). Salicylic Acid, a multifaceted hormone to combat disease. Annu. Rev. Phytopathol..

[59] Zhang Y., Yang Y., Fang B., Gannon P., Ding P., Li X., Zhang Y. (2010). Arabidopsis snc2-1D activates receptor-like protein-mediated immunity transduced through WRKY70. Plant Cell.

[60] Sun Y., Zhu Y.-X., Balint-Kurti P.J., Guan-Feng W. (2020). Fine-tuning immunity: Players and regulators for plant NLRs. Trends Plant Sci..

[61] Wang G., Roux B., Feng F., Guy E., Li L., Li N., Zhang X., Lautier M., Jardinaud M.F., Chabannes M. (2015). The decoy substrate of a pathogen effector and a pseudokinase specify pathogen-induced modified-self recognition and immunity in plants. Cell Host Microbe.

[62] Seto D., Koulena N., Lo T., Menna A., Guttman D.S., Desveaux D. (2017). Expanded type III effector recognition by the ZAR1 NLR protein using ZED1-related kinases. Nat. Plants.

[63] Baudin M., Hassan J.A., Schreiber K.J., Lewis J.D. (2017). Analysis of the ZAR1 immune complex reveals determinants for immunity and molecular interactions. Plant Physiol..

[64] Wang J., Wang J., Hu M., Wu S., Qi J., Wang G., Han Z., Qi Y., Gao N., Wang H.W. (2019). Ligand-triggered allosteric ADP release primes a plant NLR complex. Science.

[65] Wang J., Hu M., Wang J., Qi J., Han Z., Wang G., Qi Y., Wang H.W., Zhou J.M., Chai J. (2019). Reconstitution and structure of a plant NLR resistosome conferring immunity. Science.

[66] Hu G., Richter T.E., Hulbert S.H., Pryor T. (1996). Disease lesion mimicry caused by mutations in the rust resistance gene rp1. Plant Cell.

[67] Bolus S., Akhunov E., Coaker G., Dubcovsky J. (2020). Dissection of cell death induction by wheat stem rust resistance protein Sr35 and its matching effector AvrSr35. Mol. Plant Microbe Interact. MPMI.

[68] Slootweg E.J., Spiridon L.N., Martin E.C., Tameling W.I.L., Townsend P.D., Pomp R., Roosien J., Drawska O., Sukarta O.C.A., Schots A. (2018). Distinct roles of non-overlapping surface regions of the coiled-coil domain in the potato immune receptor Rx1. Plant Physiol..

[69] Tameling W.I., Nooijen C., Ludwig N., Boter M., Slootweg E., Goverse A., Shirasu K., Joosten M.H. (2010). RanGAP2 mediates nucleocytoplasmic partitioning of the NB-LRR immune receptor Rx in the Solanaceae, thereby dictating Rx function. Plant Cell.

[70] Li J., Huang H.N., Zhu M., Huang S., Zhang W.H., Dinesh-Kumar S.P., Tao X.R. (2019). A plant immune receptor adopts a two-step recognition mechanism to enhance viral effector perception. Mol. Plant.

[71] Larkin M.A., Blackshields G., Brown N.P., Chenna R., McGettigan P.A., McWilliam H., Valentin F., Wallace I.M., Wilm A., Lopez R. (2007). Clustal W and Clustal X version 2.0. Bioinformatics.

[72] Tamura K., Stecher G., Peterson D., Filipski A., Kumar S. (2013). MEGA6: Molecular Evolutionary Genetics Analysis version 6.0. Mol. Biol. Evol..

