# Supplementary material for: Maize ZmFNSI Homologs Interact with an NLR Protein to Modulate Hypersensitive Response
Source: Int J Mol Sci. 2020 Apr 5;21(7):2529. doi: 10.3390/ijms21072529 (PMC7177559; doi:10.3390/ijms21072529)
Supplement: Supplementary file 1 [file ijms-21-02529-s001.pdf]

Figure S1

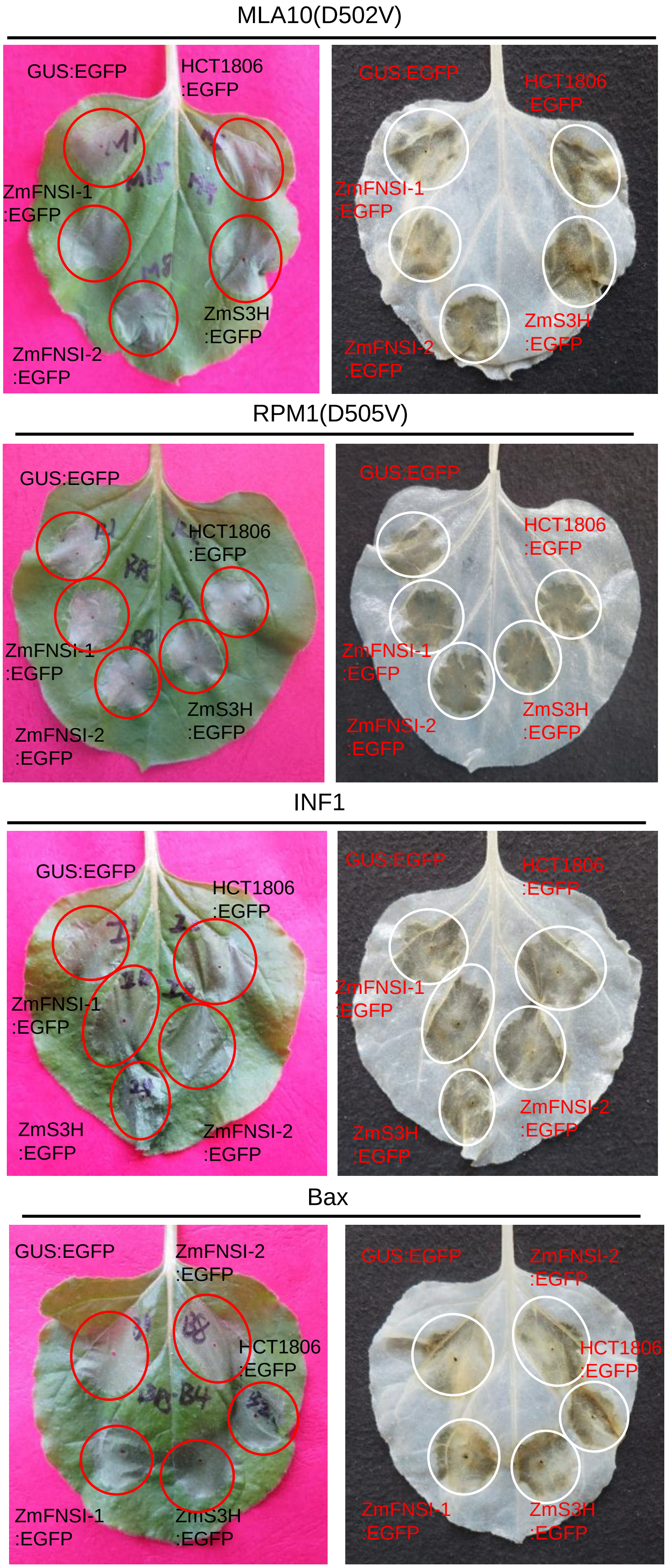

Figure S1. Investigating the function of ZmFNSIs and ZmS3H in other elicitor-mediated HR. ZmFNSIs have no obvious suppressive roles on MLA(D502V)-, RPM1(D505V)-, INF1- or Bax-mediated HR. GUS, HCT1806, ZmFNSIs and ZmS3H were transiently co-expressed with RPM1(D505V), MLA(D502V), INF1 or Bax into *N. benthamiana*. The representative leaf was photographed at 3 days after inoculation.

Figure S2

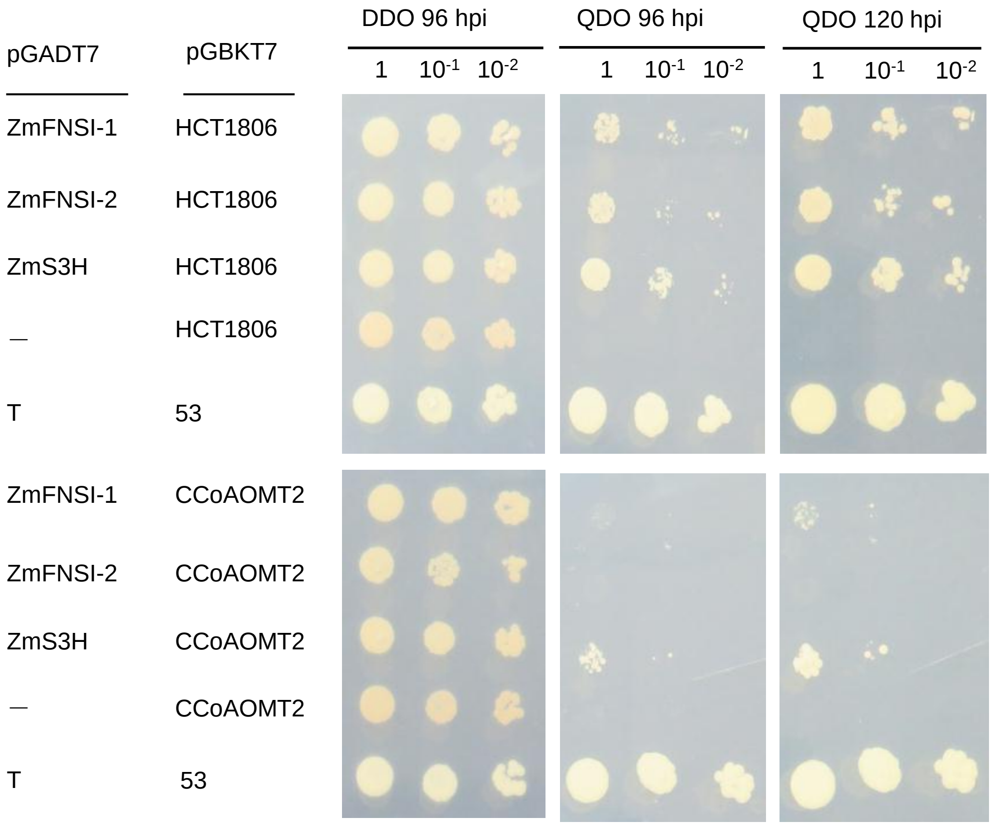

Figure S2. Investigating the interactions between HCT1806 or CCoAOMT2 and ZmFNSIs or ZmS3H. HCT1806 and CCoAOMT2 were constructed into pGBKT7 and ZmFNSIs and ZmS3H were constructed into pGADT7. T + 53 was used as the positive control. "–" indicated empty vector.

Figure S3

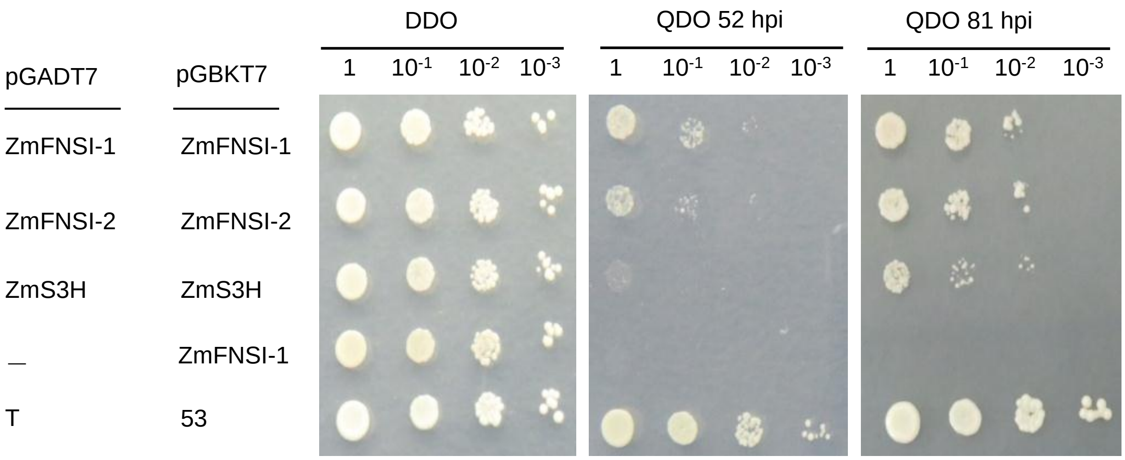

Figure S3. Investigating the self-association of ZmFNSIs and ZmS3H. ZmFNSIs and ZmS3H were constructed into pGADT7 and pGBKT7 vectors. T + 53 was used as the positive control. “－” indicated empty vector.

Figure S4

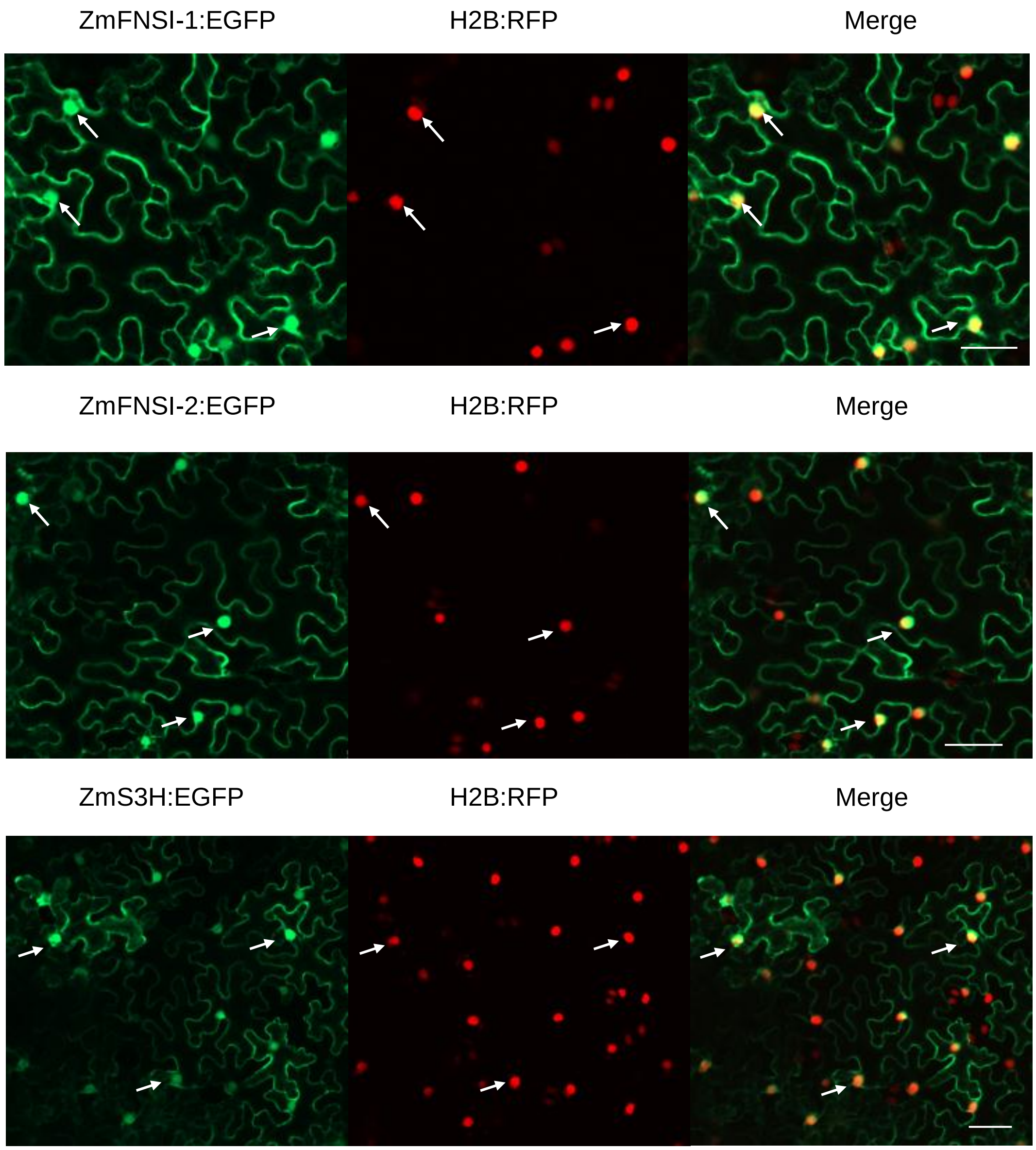

Figure S4. The subcellular localization of ZmFNSIs and ZmS3H. ZmFNSIs and ZmS3H were fused with C-terminal EGFP, and infiltrated into *N. benthamiana* transformed with nuclear marker H2B-TaqRFP. Confocal images were taken at 48 hpi. The position of the nucleus was labeled by arrows. The scale bar represents 50 μm. The experiment was repeated three times with the same results.

Figure S5

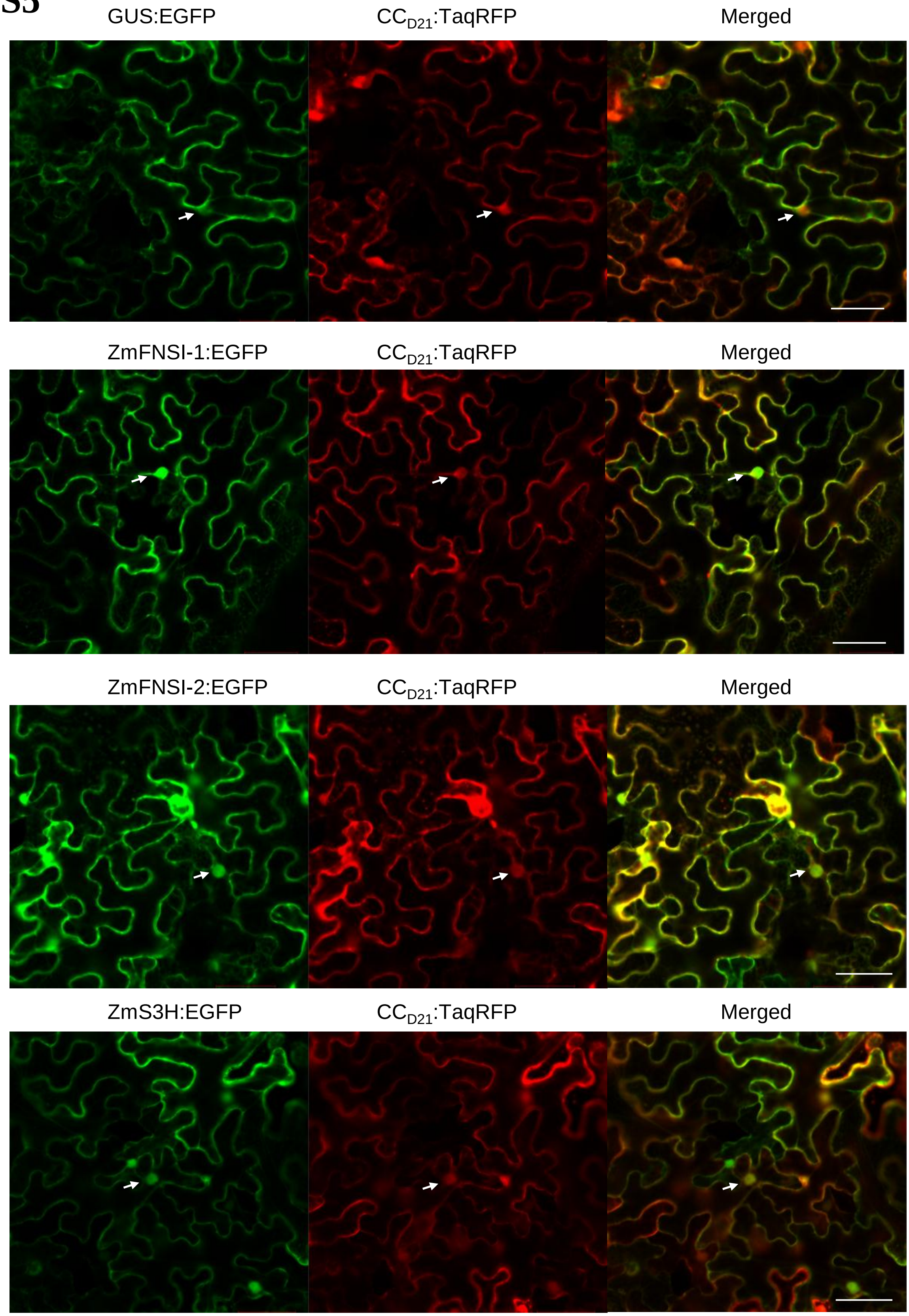

Figure S5. ZmFNSIs and ZmS3H did not change the subcellular localization of CC_{D21}. ZmFNSIs:EGFP or ZmS3H:EGFP were co-infiltrated with CC_{D21}:TaqRFP into *N. benthamiana*, and confocal images were taken at 48 hpi. The positions of the nuclei were labeled by arrows. The scale bar represents 50 μm. The experiment was repeated three times with the same results.

Table S1. The primers used in this study.

Primer name	Primer sequence（5'-3'）	Usage of the primers
ZmFNSI-1-F1	ATGGCGGAGCACCTCCTG	Amplification of ZmFNSI-1
ZmFNSI-1-R1	GGTTCTGAAGAGCTCGAGGC	
ZmFNSI-2-F1	ATGGCAGAGCATCTCATCTC	Amplification of ZmFNSI-2
ZmFNSI-2-R1	GGAGGTCGGGGTCCTGAAG	
ZmS3H-F2	GCTAACACTGCAGCCCCAAG	Nested PCR amplification of ZmS3H
ZmS3H-F1	TATAACATGGCCCCAGCC	
ZmS3H -R1	CTCCAACGATCCTCCGGG	
ZmFNSI-1-H211Q-F1	CTCCCGGCGCAAACCGAC	For constructing ZmFNSI-1(H211Q) by overlapping PCR
ZmFNSI-1-H211Q-R1	GTCGGTTTGCGCCGGGAG	
ZmFNSI-1-H268D-F1	TGTGGGACCGCGCGGTG	For constructing ZmFNSI-1(H268D) by overlapping PCR
ZmFNSI-1-H268D-R1	CACCGCGCGGTCCCACA	
ZmFNSI-2-H211Q-F1	TGCCCGCGCAAACGGAC	For constructing ZmFNSI-2(H211Q) by overlapping PCR
ZmFNSI-2-H211Q-R1	GTCCGTTTGCGCGGGCA	
ZmFNSI-2-H268D-F1	AGCGTGTGGGACCGCGC	For constructing ZmFNSI-2(H268D) by overlapping PCR
ZmFNSI-2-H268D-R1	GCGCGGTCCCACACGCT	
Zm00001d043988-F1	ATGGCGGCTAATCTCAAGTGG	Amplification of Zm00001d043988
Zm00001d043988-R1	GACGCTGCCCTTCACCTG	
Zm00001d027325-F1	ATGGGCGTCAAGCAGGTC	Amplification of Zm00001d027325
Zm00001d027325-R1	ATCTCTGGCCGCCTTGTTTC	
Zm00001d053695-F1	CACCATGGCTCCCCCGCCTCA	Amplification of Zm00001d053695
Zm00001d053695-R1	CCGCCCGACCATCGAAGA	
Zm00001d003019-F1	CACCATGCCCAAAGTTAGGATATAT CCAG	Amplification of Zm00001d003019
Zm00001d003019-R1	TCGTGGTACACGCGAGG	
Zm00001d003021-F1	CACCATGAAAGTGGTCTCTATACACC TTG	Amplification of Zm00001d003021
Zm00001d003021-R1	CCTCGCATAGTACGAACTTCTTG	
Zm00001d033460-F1	ATGGTGTCGTGGAAGAAGAAGC	Amplification of Zm00001d033460
Zm00001d033460-R1	GGCTGACGAGCTGATGATGC	
Zm00001d031666-F1	ATGGCTGCCGCTCTCGT	Amplification of Zm00001d031666
Zm00001d031666-R1	AAATTTCCATGAAGATAACGGTGG	
Zm00001d028167-F1	ATGTCGCGGCTCCTCCTCC	Amplification of Zm00001d028167
Zm00001d028167-R1	CGCACCCTGCTGCCAGC	
MTHFR2-F1	ATGAAGGTTATCGAGAAGATCCTGG	Amplification of MTHFR2
MTHFR2-R1	GATCTTGAAGGCAGCAAACAGG	

Zm00001d023843-F1	ATGGCTCCGCCTTCCTCC	Amplification of Zm00001d023843
Zm00001d023843-R1	TGGTATCGGTACAAGCCTTGG	
Zm00001d052525-F1	CTCAGAGCAATGGAGGTGGAGG	Amplification of GRMZM2G439311
Zm00001d052525-R1	GCTTGGCTGCGGCAGGGAC	
Zm00001d004916-F1	AGAGGAATGGAGGTGGAGGC	Amplification of Zm00001d004916
Zm00001d004916-R1	TCCTCTCAAGAATCGCCTTC	
Zm00001d043258-F1	ATGATGGTGAGGAAGGTAGGG	Amplification of Zm00001d043258
Zm00001d043258-R1	ATATTGAAACATCATGTGTCGCTC	
Zm00001d014126-F1	GACGAGGAGGAAATGGCG	Amplification of Zm00001d014126
Zm00001d014126-R1	CGTACATCGCACACGTATTC	
Zm00001d017425-F1	GCCGCCAACCACCATATG	Amplification of Zm00001d017425
Zm00001d017425-R1	CTCCGCCTTGGCCCTGG	
Zm00001d011081-F1	CAGGCAGCAGCCATGTCC	Amplification of Zm00001d011081
Zm00001d011081-R1	AGCAGACTCCGACTTGGTG	
